# Color-Tunable Lead Halide Perovskite Single-Mode Chiral Microlasers with Exceptionally High *g*_lum_

**DOI:** 10.1021/acs.nanolett.4c03838

**Published:** 2024-10-03

**Authors:** Haotian Gu, Haoyuan Xu, Chao Yang, Yifan Feng, Guanfeng Gao, Robert L. Z. Hoye, Xiaowen Hu, Lakshminarayana Polavarapu, Guofu Zhou, Xiao-Fang Jiang

**Affiliations:** † Guangdong Basic Research Center of Excellence for Structure and Fundamental Interactions of Matter, Guangdong Provincial Key Laboratory of Quantum Engineering and Quantum Material, School of Physics, South China Normal University, Guangzhou 510006, China; ‡ SCNU-TUE Joint Lab of Device Integrated Responsive Materials (DIRM), National Center for International Research on Green Optoelectronics, South China Academy of Advanced Optoelectronics, South China Normal University, Guangzhou 510006, China; § Inorganic Chemistry Laboratory, Department of Chemistry, University of Oxford, South Parks Road, Oxford, OX1 3QR, U.K.; ∥ CINBIO, Universidade de Vigo, Materials Chemistry and Physics Group, Department of Physical Chemistry Campus Universitario As Lagoas, Marcosende 36310, Vigo, Spain

**Keywords:** Chiral lasing, Perovskite microrods, WGM microcavity, Color-tunable, Single mode

## Abstract

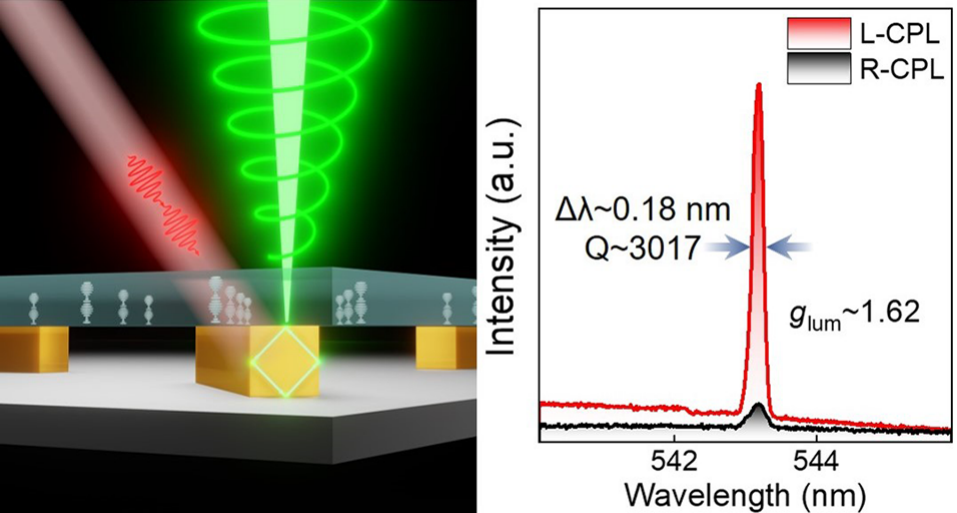

Chiral microlasers hold great promise for optoelectronics from integrated photonic devices to high-density quantum information processing. Despite significant progress in lead-halide perovskite emitters, chiral lasing with high dissymmetry factors (*g*_lum_) has not yet been realized. Here, we demonstrate chiral single-mode microlasers with exceptional stability and tunable emission across the visible range by combining CsPbCl_x_Br_3-x_ perovskite microrods (MRs) with a cholesteric liquid crystal (CLC) layer. The MRs lase via a whispering gallery mode (WGM) microcavity and confer chirality through the encapsulated CLC layer, thus exhibiting circularly polarized lasing with dissymmetry factors reaching 1.62. Importantly, we demonstrate wavelength-tunable high dissymmetry chiral lasers in a broad spectral range by tuning the halide composition and using CLC layers with the desired photonic bandgap (PBG). This facile approach to generate chiral lasing not only is applicable to semiconductor nano- and microcrystals but also paves the way for potential integration into nanoscale photonic devices.

Microlasers have garnered significant attention due to their ultrasmall dimensions, fast modulation ability, waveguiding nature, light confinement, and localized coherent emission.^1−5^ These attributes render microlasers promising for next-generation photonic and optoelectronic applications, including on-chip optical computing and information processing,^6−10^ all optical switches,^11−13^ sub-diffraction-limit imaging,^14^ optical sensing,^15^ and probing biological systems.^16,17^ A notable limitation lies in the predominance of linearly polarized microlasers. Circularly polarized (CP) microlasers are highly desired for high-density quantum information processing and computing,^18−21^ biological tissue resolution,^22,23^ and chiral sensing.^24,25^ CP lasers carry additional information in terms of optical spin that can be modulated to store or process more information. To generate chiral emission in microintegrated photonic devices that process and transmit information, chiral photonic circuits have been used by modulating the helicity and directional emission of quantum emitters.^26−28^ The use of a micro dimensional chiral light source instead of photonic circuits is more conducive for better integration into photonic devices. However, there are no effective strategies for realizing chiral microlasers, and this is one of the outstanding challenges in the field.

Recently, metal halide perovskites have emerged as a highly promising gain medium owing to their interesting optical properties including high absorption coefficient, elevated radiative recombination efficiencies, low trap densities, long charge carrier lifetimes, high photoluminescence quantum yield (PLQY), and tunable emission color by halide composition.^8,29−33^ However, the inherent centrosymmetric chemical structures of perovskite crystals preclude natural chirality, thereby limiting their direct application in chirality-associated contexts. Methods to induce chirality in perovskite nanocrystals (PNCs) include their surface functionalization with chiral ligands or arranging them into helical or twisted architectures using helical templates.^34^ However, the effectiveness, quantified by the dissymmetry factor *g*_lum_ (where *g*_lum_ = 2[*I*_L_ – *I*_R_]/[*I*_L_ + *I*_R_]; *I*_L_ and *I*_R_ are the intensities of left- and right-handed CP emission, respectively), is relatively low. The typical *g*_lum_ of chiral ligand-capped PNCs is in the range of 10^–4^ to 10^–3^,^35,36^ while the chiral assemblies exhibit slightly higher values, up to 0.1–0.3.^37−39^ Notably, a few studies have demonstrated high *g*_lum_ (up to 1.9) by combining PNCs with CLCs, in which the CLCs selectively reflect one specific polarization, leading to CP emission with high efficiency.^40^ Toward this direction we recently demonstrated CP-amplified spontaneous emission (ASE) from layers of PNCs sandwiched between CLCs in a Fabry–Perot (F–P) cavity structure, achieving a *g*_lum_ of 1.4.^41^ Despite significant progress in improving the CP-emission efficiency and achieving CP-ASE, chiral microlasers based on perovskite nano- and microcrystals (also other types of semiconductors) have not yet been realized and remain a formidable challenge.

Here we introduce a facile approach to achieve single-mode circularly polarized lasing (CPL) from perovskite microrods (MRs) by encapsulation with a layer of CLCs. We chose the perovskite MRs with a rectangular cross-section that has an internal WGM microcavity to exhibit lasing, yielding an exceptional *Q*-factor of 3017. By incorporating the CLC layer, chirality is induced through selective Bragg reflection, resulting in CPL with a *g*_lum_ up to 1.62 for CsPbBr_3_ MRs (Figure 1a). Crucially, by controlling the halide (Br to Cl) composition and adjusting the PBG of CLCs to the corresponding wavelength, we demonstrate wavelength-tunable chiral single-mode microlasers from deep blue to pure green with *g*_lum_ ∼ 1.60. The microlasers exhibit exceptional stability under continuous optical pumping and show long-term stability under ambient conditions due to the protective properties conferred by both the CLC layer and substrate.

**Figure 1:**
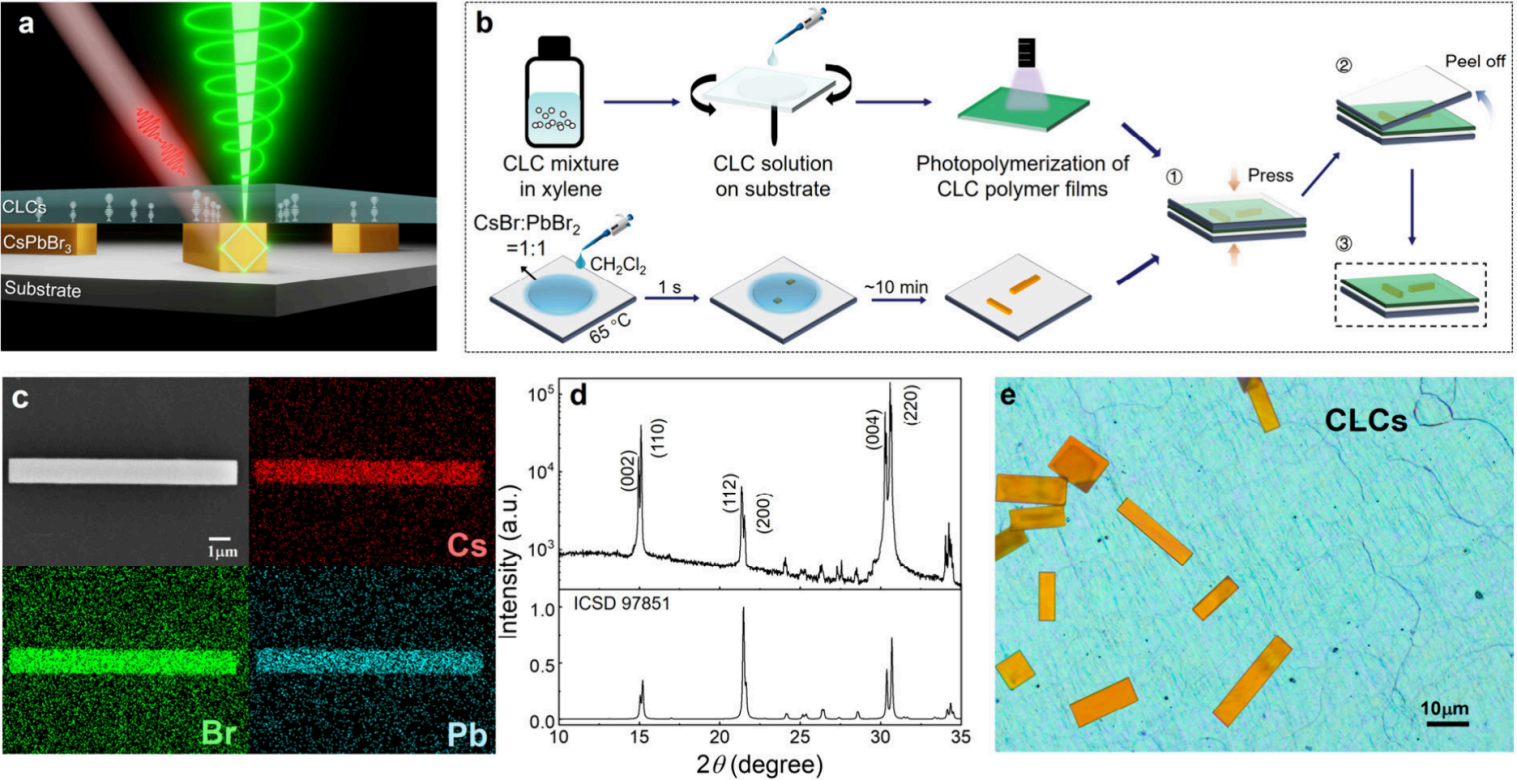
a) Schematic of the architecture used to achieve a chiral microlaser, showing the device cross-section and the mechanism of chiral lasing, in which the perovskite MRs are sandwiched between the substrate and a layer of CLCs, where the CLCs selectively reflect one specific polarization of linearly polarized lasing emission generated from the perovskite MRs. b) Fabrication process of the CsPbBr_3_ and CLC composite device. c) SEM image and EDS mapping of CsPbBr_3_ MRs. d) X-ray diffraction (XRD) patterns and standard card (ICSD 97851). e) Bright-field POM image using focal stacking.

The composite comprising CLCs/perovskite MRs/substrate is specifically engineered to achieve highly anisotropic chiral microlasers (Figure 1a). The inset illustrates the methodology employed to obtain chiral microlasers. The lasing is facilitated by WGM within the cross-section of the CsPbBr_3_ MRs. The transition from typical linearly polarized lasing to CPL is realized through the utilization of a CLC film with helical photonic crystal structures. First, the CsPbBr_3_ MRs were fabricated via an ultrafast one-step antisolvent-induced recrystallization method (Figure 1b, see the Methods for synthesis details).^42,43^ The typical scanning electron microscopy (SEM) image of CsPbBr_3_ MRs and the energy dispersive X-ray spectroscopy (EDS) mapping of Cs, Pb, and Br atoms are shown in Figure 1c, demonstrating the uniform distribution of each element within the prepared MRs. Meanwhile, its structure is characterized by X-ray diffraction (XRD) as shown in Figure 1d, which suggests that the prepared CsPbBr_3_ MRs belong to the orthorhombic phase. Subsequently, a CLC mixture was blade-coated onto silica glass and polymerized into a film under UV irradiation. The CLC/perovskite MR layer-by-layer structure was then obtained by sandwiching CLC films with CsPbBr_3_ MRs followed by the removal of the top glass (Figure 1b). Figure 1e shows the polarizing optical microscopy (POM) image of the perovskite MRs between the glass substrate and the CLCs. We used a technique called focus stacking to portray multiple objects in focus on various focal planes in one sharp image, where everything is in focus (Figure S1a). The original image, with a direct focus on the underlying CLC, is shown in Figure S1b. The presence of a typical oily streak texture as well as the obvious reflective band in Figure S1c serve as evidence for the formation of chiral structures.

To demonstrate a high dissymmetry chiral microlaser using the CLC/perovskite MR architecture, the CsPbBr_3_ MRs act as a gain medium with an internal WGM cavity. Figure 2a displays a cross-sectional SEM of a CsPbBr_3_ MR, showing a rectangular cross-section. Power-dependent photoluminescence (PL) spectra of the CsPbBr_3_ MRs were acquired under two-photon pump excitation at 800 nm (Figure 2b). Below the threshold (*P*_Th_), only a broad spontaneous emission peak at a wavelength of 533 nm was observed, which is typical for CsPbBr_3_ MRs.^2,44^ Upon exceeding the *P*_Th_, a sharp peak at around 543 nm emerged and intensified significantly with a further increase of pump fluence. Figure 2c depicts the PL intensity and full width at half-maximum (fwhm) as a function of pump fluence. A noticeable inflection point is observed with the increase in pump fluence, indicating the transition from spontaneous emission to stimulated emission at *P*_Th_ ≈ 94.2 μJ cm^–2^. The lasing wavelengths and thresholds remain consistent (with only minor variations) in different CsPbBr_3_ MRs of a similar rectangular cross-section (see Figure S2). Finite-difference time-domain (FDTD) simulations were conducted to analyze the resonant cavity in the transverse plane of the CsPbBr_3_ MRs. The FDTD simulation depicted in Figure 2d shows the electric fields of the fundamental mode within the MRs, confirming that the lasing originates from the WGM within the cross-section of the CsPbBr_3_ MRs. The TE mode number (*N*) of the WGM microcavity in the square cross-section is derived as , where *L* is the length of the cavity, *n* is the refractive index, and λ is the resonant wavelength.^44^ As the cavity size reduces to ≈2 μm, the single-mode lasing output is formed due to the existence of the self-absorption effect and the enlarged mode spacing.^1,45^ The perovskite MRs are selectively excited to obtain a single-mode CPL in subsequent devices.

**Figure 2:**
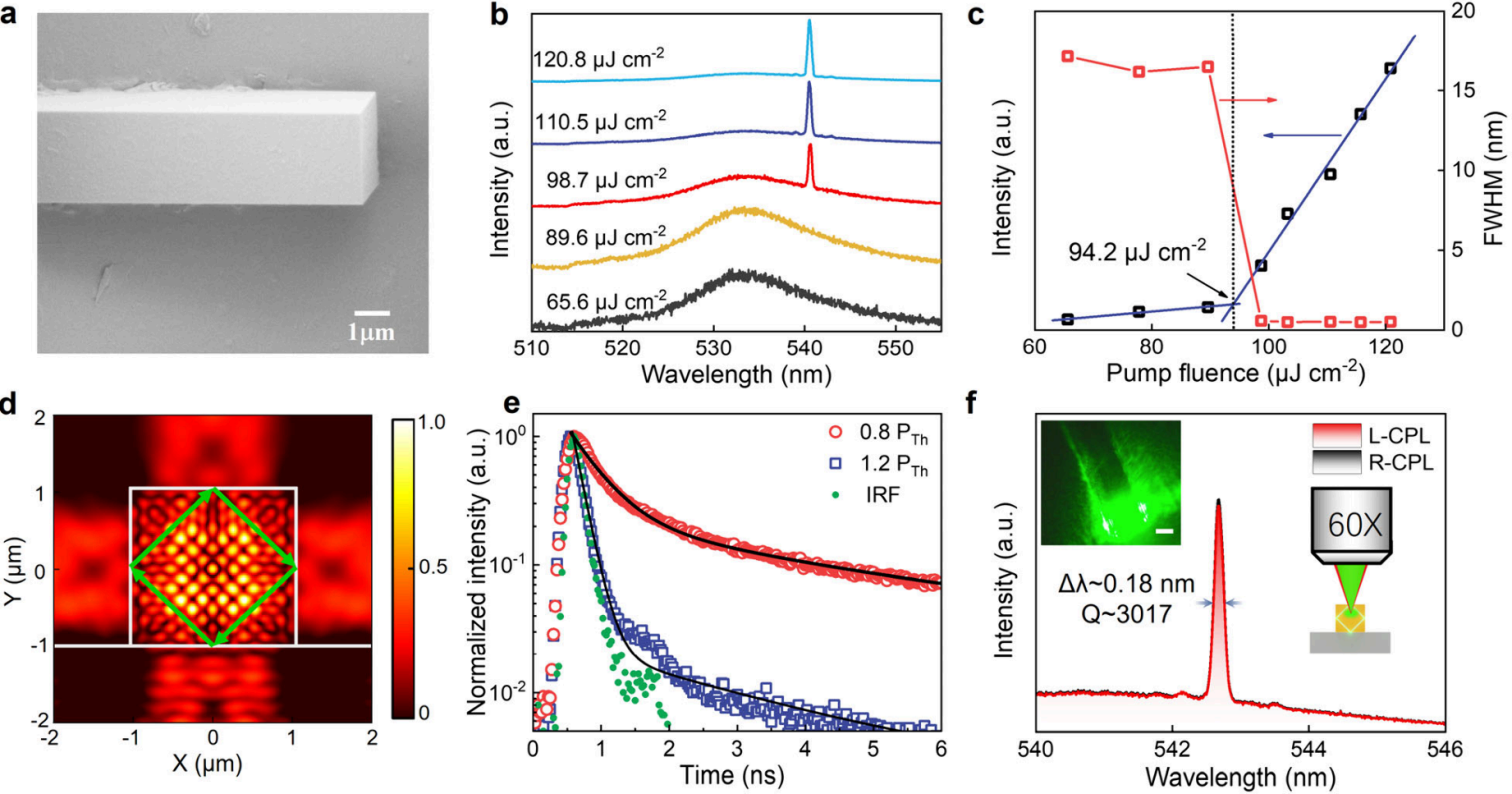
a) SEM image of the CsPbBr_3_ MR. b) Evolution of PL spectra of the MR with the increase of pump fluence. c) Pump-power-dependent PL intensity and fwhm of the CsPbBr_3_ MR, showing lasing characteristics from the excitation fluence of 94.2 μJ cm^–2^. d) FDTD simulations showing the electric-field distribution in the CsPbBr_3_ MR. e) Time-resolved PL decay of CsPbBr_3_ at two different excitation densities (0.8*P*_Th_ and 1.2*P*_Th_) (IRF: instrument response function). f) The PL spectra of the CsPbBr_3_ MR probed with L- and R-CP polarizers, and exhibiting a quality factor of 3017. Inset: optical image of CsPbBr_3_ lasing (scale bar: 1 μm).

Further investigations into the charge carrier dynamics of CsPbBr_3_ MRs were garnered through time-resolved photoluminescence (TRPL) measurements. Figure 2e depicts typical PL decay curves of CsPbBr_3_ MRs below and above the threshold. At a low pump fluence (0.8*P*_Th_), a biexponential decay function with rapid and slow components was employed to model the decay curves. A marginal disparity in lifetimes was observed at 0.8*P*_Th_ (451.2 ps, 63.4%; 1601.9 ps, 36.6%), indicating the presence of a fraction of non-radiative recombination alongside predominantly radiative recombination at such low pump fluences.^8,46^ Upon increasing the pump fluence to 1.2*P*_Th_, an additional decay component emerges (258.1 ps, 96.9%), which dominates over the other two components (552.3 ps, 1.7%; 1558 ps, 1.3%), suggesting the onset of an effective stimulated emission process.

To examine the polarization of the microlaser without the CLC layer, the emission was analyzed using left-circularly polarized (L-CP) and right-circularly polarized (R-CP) filters composed of a λ/4-waveplate and a polarizer placed in front of the optical fiber used for light collection (see the schematic setup illustration in Figure S3). The acquired lasing spectra of the CsPbBr_3_ MR with both polarizations are depicted in Figure 2f. The shape of the spectra and the intensity for both L-CP and R-CP lasing were identical, indicating the linear polarization of the emission. The fwhm of the peak is measured to be 0.18 nm, and the quality factor (*Q*-factor) was calculated to be ≈3017 (*Q* = λ/δλ, where λ represents the peak center wavelength and δλ denotes the fwhm of the peak). Additionally, as depicted in Figure S4, the polarization-dependent lasing spectra were recorded by removing the λ/4-waveplate. The degree of polarization was determined to be as high as 88.8% according to Marussia’s law, i.e., (*I*_max_ – *I*_min_)/(*I*_max_ + *I*_min_), indicating a high degree of linear polarization.

To facilitate the transformation of linearly polarized lasing from perovskite MRs into circularly polarized lasing, a CLC layer is incorporated into the architecture (Figure 1a). The CLCs serve as the quintessential reflective material. The molecular structures of the materials used for preparing the CLC mixture are illustrated in Figure S5, drawing inspiration from our previous work.^41^ In these materials, the RM-257 acts as a diacrylate monomer capable of cross-linking into a polymer network under UV irradiation, facilitated by the photoinitiator Irgacure-651. A surfactant is employed to regulate the liquid crystal alignment at the air interface and prevent dewetting during the coating process. On the other hand, the LC-756 acts as a chiral monomer, inducing a right-handed helix that enables the CLC film to reflect light with right-handed polarization. The wavelength of light reflected by the CLC layer is determined by Δλ = Δ*nP* = (*n*_e_ – *n*_o_)*P*, where Δ*n* represents the birefringence index of the CLC mixture and *n*_e_ and *n*_o_ denote the extraordinary and ordinary refractive indexes, respectively. Additionally, *P* signifies the helical pitch of the CLC film, defined by the equation *P* = 1/(HTP × *C*), where HTP represents the helical twisting power of the chiral monomer and *C* denotes the concentration of the chiral monomer in the CLC mixture.^47,48^ According to Bragg’s law, as depicted in Figure 3a, the central reflection peak of CLCs is influenced by the *P*, the average refractive index (*n*_av_) of the material, and the incident angle (θ) of the light, i.e., λ(θ) = *n*_av_*P* cos θ. With an increase in the incidence angle, the PBG of CLCs undergoes a blueshift. By adjustment of the concentration of LC-756 in the CLC mixture, control over the PBG of the CLC film was attained (Table S1). Figure 3b shows the variation of the PBG of CLCs-1 with the angle of incidence and the corresponding lasing peak of CsPbBr_3_ MR. For comparison, CLCs-2 and CLCs-3, with blue-shifted and red-shifted PBGs, respectively, were employed in the laser devices (Figure S5a,b). We proceeded to investigate the CPL properties of the composite device. Parts c and d of Figure 3 present the lasing performance of the device made with CLCs-1 with a threshold pump fluence of approximately 81.8 μJ cm^–2^. At high pump fluences of 93.6 μJ cm^–2^, the L-CP lasing intensity significantly surpasses that of R-CP lasing, with a *g*_lum_ reaching 1.62 (Figure 3e). The *g*_lum_ remained consistent at pump fluences above the threshold (Figure S6). Similar configurations using CLCs-2 and CLCs-3 films were employed to assess CPL properties. Interestingly, the lasing wavelength coincides with the center of the normal incident PBG of CLCs-2. However, no chiral emission was observed (Figure S7c), with L-CP and R-CP lasing intensities almost identical, yielding a *g*_lum_ value close to 0.07. In contrast, the lasing wavelength of CLCs-3 deviates from the normal incident PBG. As the angle of incidence increases, the PBG of CLCs-3 gradually encompasses the lasing wavelength, resulting in chiral lasing with a *g*_lum_ of ∼0.83 (Figure S7c). Notably, for the device with CLCs-1 exhibiting the highest *g*_lum_, the lasing wavelength initially resides at the short-wave edge of the normal incident PBG, progressively moving toward the center as the angle of incidence increases. Consequently, the WGM lasing from the cross-section of CsPbBr_3_ MRs occurs at an angle across the CLC film. The CP characteristics of the lasing emission are further corroborated by recording the transmittance of the emission passing through a λ/4 waveplate followed by a rotated analyzer. The λ/4 waveplate converts CPL to linearly polarized light that can be readily examined by a linear analyzer. As shown in Figure 3f, the L-CP lasing is converted to a linearly polarized light with orthogonal plane polarization. However, upon removal of the λ/4 waveplate, the shape of the graph is elliptical instead of a perfect circle due to the small percentage of L-CPL in the emission.

**Figure 3:**
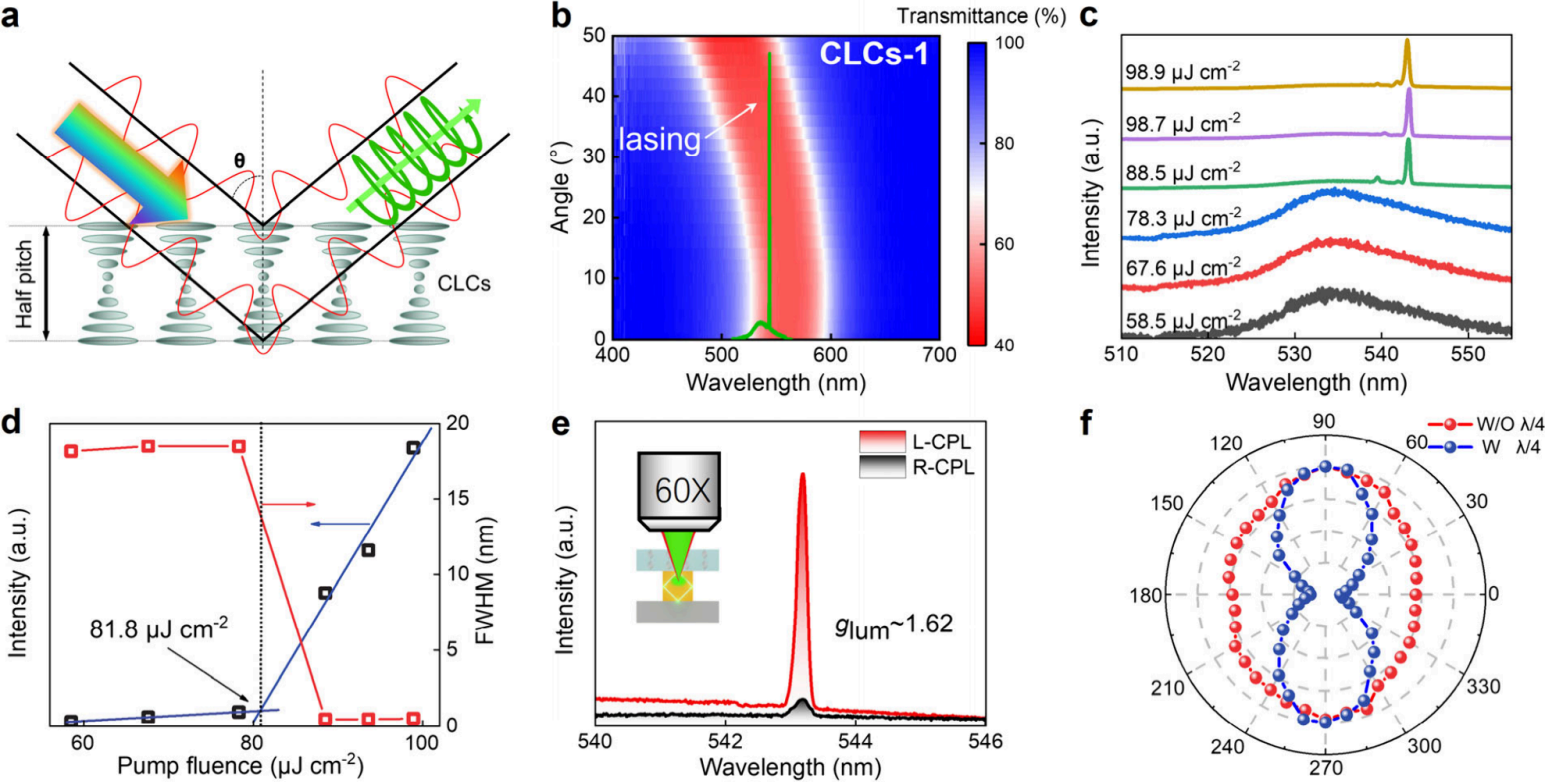
a) Bragg reflection principle of CLCs; R-CP light corresponds to the angle-dependent PBG being reflected at white light incidence. b) Incident light angular-dependent PBG of CLCs-1 and lasing spectra of CsPbBr_3_. c) Evolution of PL spectra of the device with the increase of pump fluence. d) Pump-power-dependent PL intensity and fwhm of the device. e) Measured CPL spectra of the composite device above threshold. f) Emission intensity of L-CP lasing as a function of polarization angle with and without a λ/4 waveplate in the polar coordinate system.

Regarding halide perovskite-based devices, achieving long-term stability under ambient conditions is extremely challenging due to their sensitivity to moisture as well as the low activation energy barrier to ion migration. Surprisingly, in this work, the integrated lasing intensity of the CsPbBr_3_ MR versus the excitation time shows stable performance for over 1.8 × 10^6^ lasing shots in 30 min (Figure 4a). These results suggest that the CsPbBr_3_ MRs have significantly longer operating lifetimes under ambient conditions. In addition, the device exhibits outstanding long-term stability without any additional protection, exhibiting *g*_lum_ ∼ 1.6 after a month of storage under ambient environment at a humidity of 60% and temperature of 25 °C (Figure 4b). The higher stability is attributed to the protection of perovskite MRs from CLCs and substrates that isolate the MRs from external moisture and oxygen.

**Figure 4:**
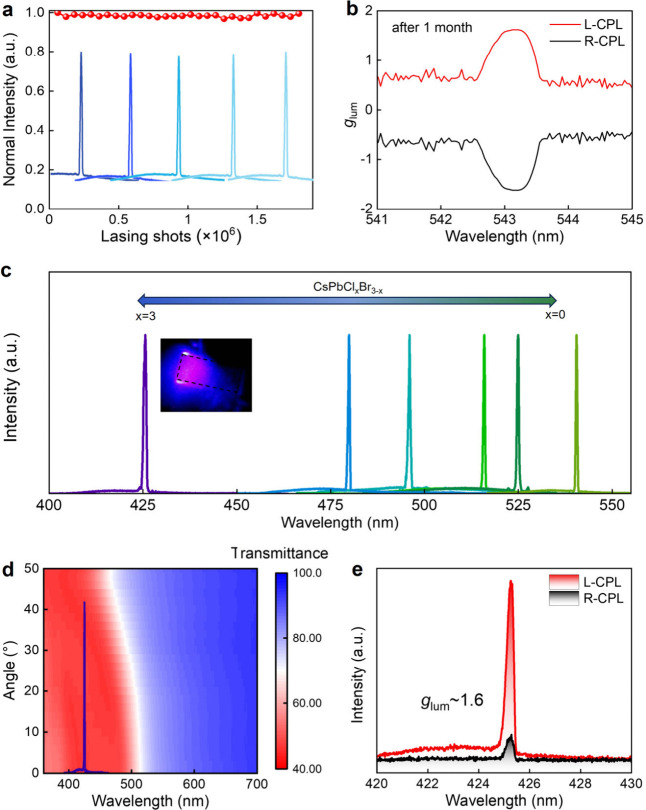
a) Operational stability showing the aging of the lasing intensity of the device under the continuous excitation of the pumped-pulse laser in the ambient environment. b) Measured *g*_lum_ spectra of L- and R-CPL after a month (room temperature, ambient atmosphere with RH of ≈60%). c) Tunable lasing spectrum of CsPbCl_*x*_Br_3–*x*_ MRs. d) Angular-dependent PBG of CLCs-4 and lasing spectrum of CsPbCl_3_. e) Measured CPL spectra of the CsPbCl_3_ composite device above threshold.

We then studied the tunability of the chiral lasing wavelength by varying the halide composition (Br to Cl) using the same device architecture. To achieve Bragg reflection conditions, the photonic bandgap of CLC was adjusted to the emission of the perovskite MRs (see the Methods). Figure 4c depicts the continuously tunable chiral lasing from ∼420 to 530 nm using CsPbCl_*x*_Br_3–*x*_ MRs of different Cl/Br ratios. To achieve a blue color chiral microlaser, blue emissive CsPbCl_3_ MRs were coupled to the CLCs-4 film in the same device architecture (Figure 4d). As the center wavelength coincides with the edge of the PBG band gap of CLCs-4, the L-CP lasing intensity again surpasses that of the R-CP lasing, with a *g*_lum_ reaching ∼1.6 (Figure 4e). This reveals the universal applicability of this approach to halide perovskites that emits different colors as well as other semiconductor materials.

## Conclusions

The results presented above demonstrate the realization of wavelength-tunable single-mode chiral microlasers with high *g*_lum_ (∼1.6) and a stable output. This is achieved through the combination of perovskite MRs and CLCs, where the perovskite MRs lase under optical pumping and the CLCs transform the linearly polarized lasing into CPL through Bragg reflection. The microlaser exhibits remarkable characteristics, including a high *Q* of 3017 and a low lasing threshold of 81.8 μJ cm^–2^. Furthermore, the device demonstrates exceptional long-term stability with constant laser output under continuous pumping owing to the protective CLC layer surrounding the perovskite MRs. The tunability of the lasing wavelength is achievable through halide composition engineering of the gain media plus the intrinsic cavity properties of the microcrystal morphology and the tunable PBG of the CLCs. A deep blue chiral microlaser with *g*_lum_ ∼ 1.60 is achieved using CsPbCl_3_ and CLCs with corresponding PBG. These outstanding features make the chiral microlasers highly promising as building blocks in next-generation on-chip computing and information processing, nanophotonics, and diagnostics. The approach reported to achieve a chiral microlaser is universally applicable to different types of semiconductor nano- and microcrystals that exhibit internal gain.

## Supporting Information Available

Methods and materials, polarized optical microscope images, lasing spectra of different MRs, schematic diagram of the optical setup for characterization of CPL, polarization-dependent emission spectra, the structure of the molecules used in preparing CLC films, angular-dependent PBG of CLCs-2 and CLCs-3, mass ratio of the molecules used to prepare CLC layers of different PBGs, and the comparison of the lasing threshold for the WGM laser of perovskite microrods or microwires (PDF)
