# Supplementary material for: Color-Tunable Lead Halide Perovskite Single-Mode Chiral Microlasers with Exceptionally High glum
Source: Nano Lett. 2024 Oct 3;24(42):13333–40. doi: 10.1021/acs.nanolett.4c03838 (PMC11503764; doi:10.1021/acs.nanolett.4c03838)
Supplement: Supplementary file 1 — nl4c03838_si_001.pdf [file nl4c03838_si_001.pdf]

Supporting Information

Color-tunable Lead Halide Perovskite Single-mode Chiral Microlasers with Exceptionally High g_{lum}

Haotian Gu[1#], Haoyuan Xu[1#], Chao Yang[2#], Yifan Feng[2], Guanfeng Gao[1], Robert L. Z. Hoye[3], Xiaowen Hu[2], Lakshminarayana Polavarapu[1,4*], Guofu Zhou[2], Xiao-Fang Jiang[1*]*

[1]Key Laboratory of Atomic and Subatomic Structure and Quantum Control (Ministry of Education), Guangdong Provincial Key Laboratory of Quantum Engineering and Quantum Material, School of Physics, South China Normal University, Guangzhou 510006, China.

[2]SCNU-TUE Joint Lab of Device Integrated Responsive Materials (DIRM), National Center for International Research on Green Optoelectronics, South China Academy of Advanced Optoelectronics, South China Normal University, Guangzhou 510006, China.

[3]Inorganic Chemistry Laboratory, Department of Chemistry, University of Oxford, South Parks Road, Oxford, OX1 3QR, UK.

[4]CINBIO, Universidade de Vigo, Materials Chemistry and Physics Group, Department of Physical Chemistry Campus Universitario As Lagoas, Marcosende 36310, Vigo, Spain.

Methods

Synthesis of $CsPbCl_xBr_{3-x}$ MRs: The $CsPbBr_3$ precursor solutions with a molar concentration of 0.04 M were prepared by dissolving CsBr and $PbBr_2$ in the molar ratios of 1:1 in DMF and stirring at 70 °C for 12h ($CsPbCl_3$: CsCl:$PbCl_2$ = 1:1 saturated solution in DMF). The substrate was cleaned by ultrasonic agitation for 5 min each in alcohol, acetone, and DI water, which was then placed on a hot plate at 65°C. Fifty microliters of precursors were dropwise added onto the substrate. After a drop of antisolvent CH_2Cl_2 (10 μL), a large number of crystal nuclei were formed in several seconds, which grew into MRs within the next 10 min. For the synthesis of $CsPbCl_xBr_{3-x}$, all the processes were the same under the corresponding ratios of Cl to Br.

Preparation of CLCs polymer films: The CLCs polymer film was prepared by blade-coating, as depicted in Figure 1b. RM-257 (93 wt%), chiral dopant of LC-756 (5 wt%), Irgacure 651 (1 wt%) and 2-n-ethylperfluoro-octanesulfonamido-ethyl methacrylate (1 wt%) were dissolved in 1.5 wt% of xylene under constant stirring at 50 °C for 1 h to obtain the CLC mixture. Subsequently, the mixed solution was blade-coated on pre-prepared glass substrates, and then polymerized under the illumination of UV light (7.5 mW/cm^2) for 5 min, thus yielding a CLCs polymer film. The thickness of the CLCs film was controlled by the gap between the coating bar and substrate.

Fabrication of Composite Device: as shown in Figure 1b, after the synthesis of $CsPbBr_3$ MRs was completed, CLCs film was covered with MRs and pressure was applied to them for 10 min. Previously, the CLCs was scraped down to form a flexible film. Then, the glass of the CLCs film is removed to form the $CsPbBr_3$ MRs-liquid crystal composite device.

Steady-state lasing emission spectra: Steady-state lasing emission spectra was acquired with a Princeton Instruments SP2150 Spectrometer. The output port was integrated with a CCD (PIXIS100) for Spectrum collecting. $CsPbBr_3$ MRs and devices were pumped by an 800 nm 200 fs laser pulses with a repetition of 1kHz through a 50× objective lens, while the polarization of the emission was analyzed by placing a left or right circular polarization filter, which is consisted of a λ/4 waveplate and a polarizer, in front of the collection optical fiber. The optical setup was illustrated in Figure 2g.

Time-Resolved Photoluminescence (TRPL): Time resolved PL spectroscopy was performed by a home-build system, which mainly includes fs pulsed laser and a spectrometer integrated with Time Correlated Single Photon Counting (TCSPC) detection. The excitation laser pulses were generated from a wavelength-tunable femtosecond laser with 200 fs pulse width and a repetition rate of 25 KHz. The excitation laser was focused on the sample via an objective lens (50×). The collected emission was further imported into a spectrometer (Andor Kymera-328i). The output port was integrated with a photomultiplier detector (PicoQuant PMA182) which was associated with TCSPC (HydraHarp 400) for TRPL measurement.

Scanning Electron Microscopy (SEM) and Energy Dispersive Spectroscopy (EDS)

The SEM images along with an EDS analysis were obtained from Carl Zeiss, ZEISS Gemini 500, which has a secondary electron detector and a backscattered electron detector (In-Lens). The sample was attached to a conducting substrate, and the platinum metal coating was sputtered onto the sample before measurement.

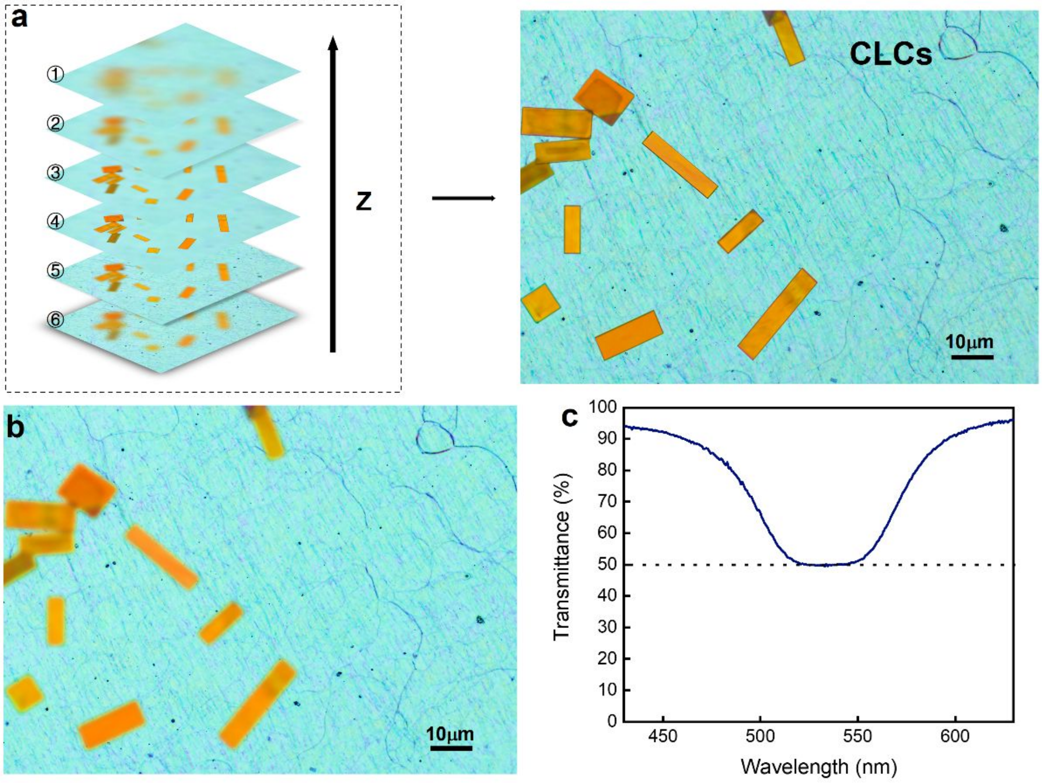

Figure S1. a) Focus stacking method by combining different focal surfaces of polarized optical microscope images. b) The fifth image of a) focused on CLCs oily streak texture. c)Transmittance spectra of CLC-1 at normal incident.

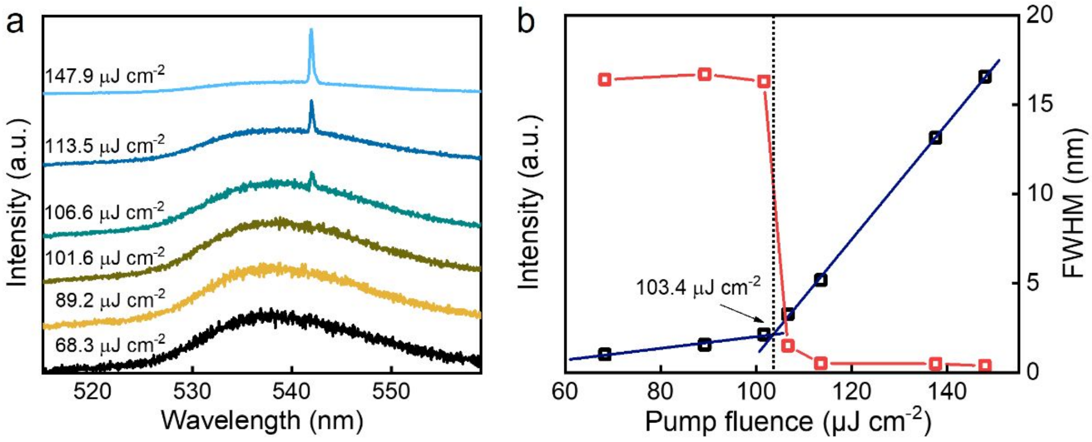

Figure S2. a) Evolution of PL spectra of different $CsPbBr_3$ MR with similar size of rectangular cross section (~2 μm side length) upon the increase of pump fluence. b) Pump power-dependent PL intensity and FWHM, showing lasing characteristics from the excitation fluence of 103.4 μJ cm^{-2}.

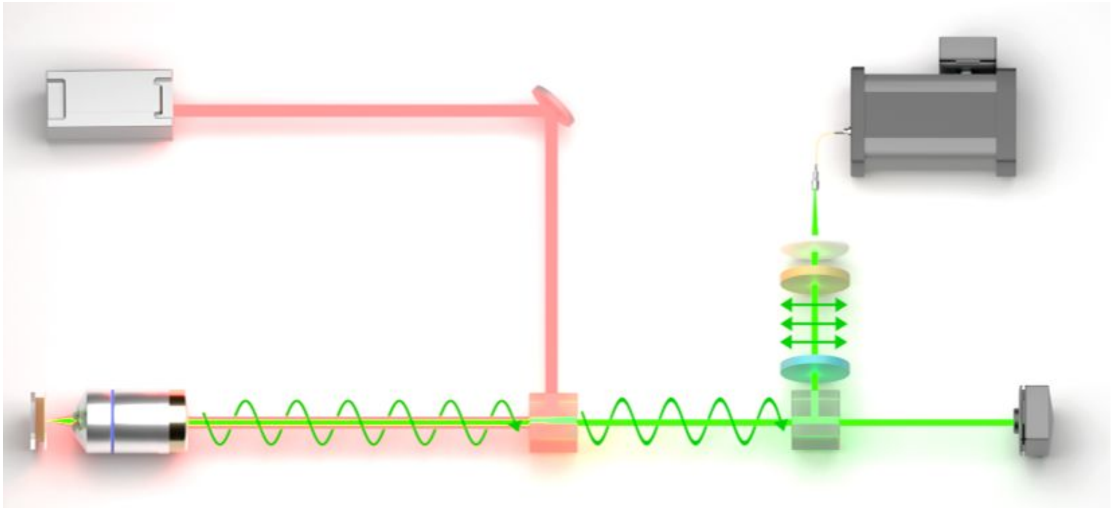

Figure S3. Schematic diagram of the optical setup for characterization of CPL.

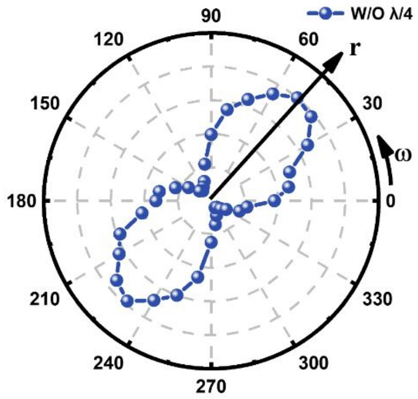

Figure S4. The polarization-dependent emission spectra for $CsPbBr_3$ MWs by removing the λ/4 waveplate.

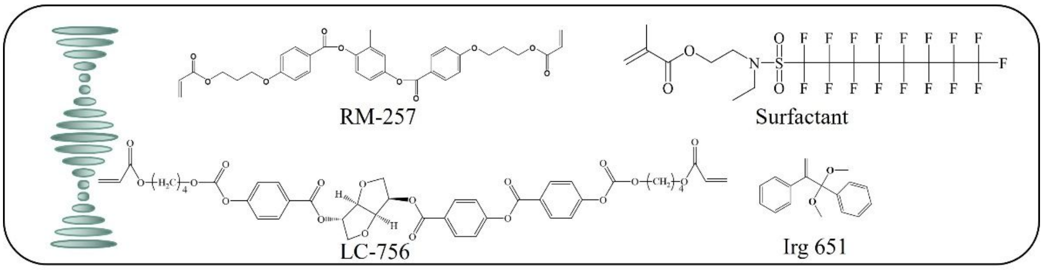

Figure S5. The structure of the molecules used in preparing CLCs films.

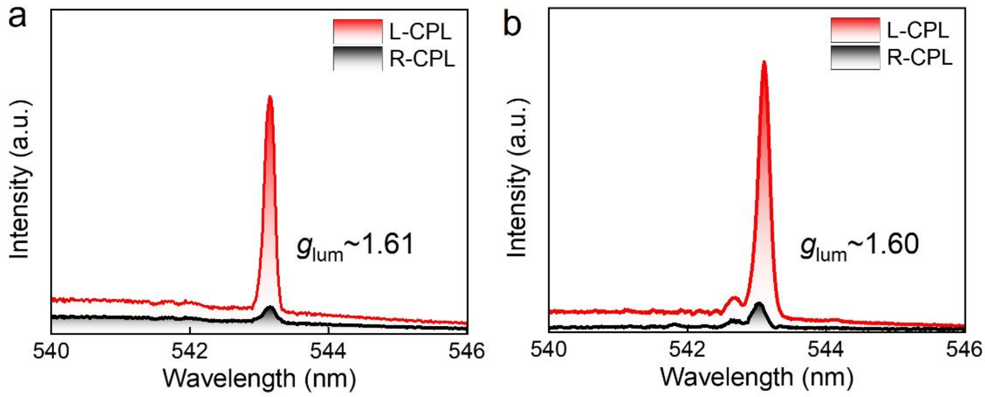

Figure S6. The measured CPL spectra of the composite device with various pump fluence of a)88.5 b) 98.9 μJ cm^{-2}.

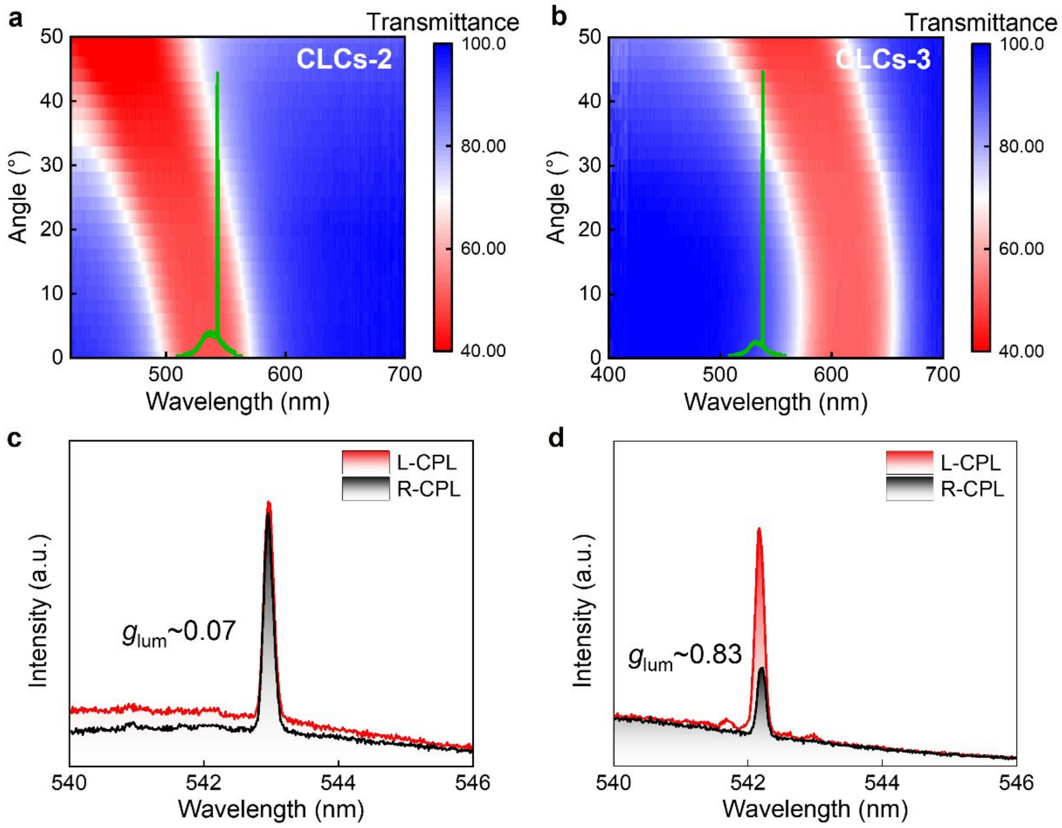

Figure S7. a) and b) Incident light angular-dependent PBG of CLCs-2 and CLCs-3 with lasing spectra of $CsPbBr_3$, respectively. c) and d) The measured CPL spectra of the composite device with CLCs-2 and CLCs-3 above threshold, respectively.

Table S1: The mass ratio of the molecules used to prepare CLCs layers of different PBGs.

	CLCs-1	CLCs-2	CLCs-3	CLCs-4
RM-257	93.3%	93%	93.7%	92.1%
LC-756	4.7%	5%	4.3%	5.9%
lrg 651	1%	1%	1%	1%
Surfactant	1%	1%	1%	1%

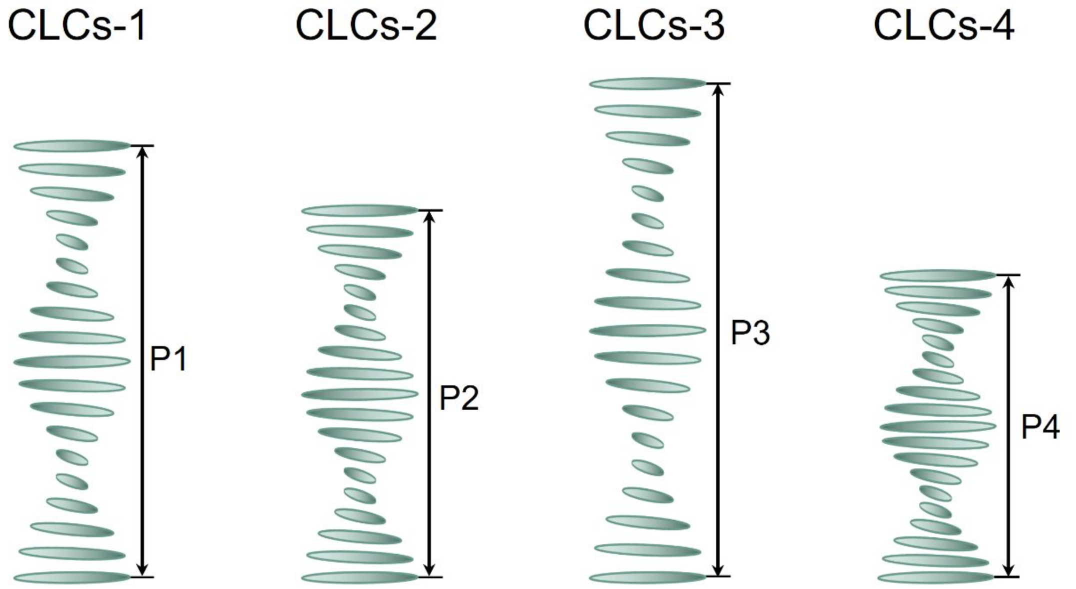

Table S2: The comparison of the lasing threshold for the WGM laser of perovskite microrods or microwires.

Material	Threshold/μJ cm^{-2}	Pump laser	ref
$CsPbBr_3$ microrods	5.2	400 nm	(1)
$MAPbBr_3$ microrods	2.37	400 nm	(2)
$CsPbBr_3$ microwires	2.2	400 nm	(3)
$CsPbBr_3$ microwires	150<br>18×10^3	355 nm<br>800 nm	(4)
$CsPbBr_3$ microwires	9.1	355 nm	(5)
$CsPbBr_3$ microrods	81.8	800 nm	This work

REFERENCES

(1) Wang, S.; Wang, K.; Gu, Z.; Wang, Y.; Huang, C.; Yi, N.; Xiao S.; Song, Q. Solution-Phase Synthesis of Cesium Lead Halide Perovskite Microrods for High-Quality Microlasers and Photodetectors. *Adv. Opt. Mater.* **2017**, *5*(11), 1700023.

(2) Wang, K.; Sun, S.; Zhang, C.; Sun, W.; Gu, Z.; Xiao, S.; Song, Q. Whispering-gallery-mode based $CH_3NH_3PbBr_3$ perovskite microrod lasers with high quality factors. *Mater. Chem. Front.* **2017**, *1*(3), 477-481.

(3) Yang, Z.; Lu, J.; ZhuGe, M.; Cheng, Y.; Hu, J.; Li, F.; Qiao, S.; Zhang, Y.; Hu, G.; Yang, Q.; Peng, D.; Liu, K.; Pan, C. Controllable Growth of Aligned Monocrystalline $CsPbBr_3$ Microwire Arrays for Piezoelectric-Induced Dynamic Modulation of Single-Mode Lasing. *Adv. Mater.* **2019**, *31* (18), 1900647.

(4) Lu, J.; He, X.; Xu, J.; Li, F.; Tang, Q.; Wang, X.; Dai, J.; Yao, Q.; Qin, F.; Xu, C. Two-Photon Pumped Single-Mode Lasing in $CsPbBr_3$ Perovskite Microwire. *Adv. Funct. Mater.* **2023**, 2308957.

(5) Li, F.; Yang, Z.; Jiang, M.; Wang, C.; Xi, J.; Zhang, Y.; Pan, C.; Lu, J.; Wang, R. Wavelength Tunable Single-Mode Lasing from Cesium Lead Halide Perovskite Microwires. *Appl. Phys. Lett.* **2021**, *118* (7), 071103.
